# Phage Displayed Short Peptides against Cells of *Candida albicans* Demonstrate Presence of Species, Morphology and Region Specific Carbohydrate Epitopes

**DOI:** 10.1371/journal.pone.0016868

**Published:** 2011-02-22

**Authors:** Soshee Anandakumar, Kannan Narayanarao Boosi, Harigopalarao Bugatha, Bhavna Padmanabhan, Parag P. Sadhale

**Affiliations:** Department of Microbiology and Cell Biology, Indian Institute of Science, Bangalore, India; Institute of Developmental Biology and Cancer Research, France

## Abstract

*Candida albicans* is a commensal opportunistic pathogen, which can cause superficial infections as well as systemic infections in immuocompromised hosts. Among nosocomial fungal infections, infections by *C. albicans* are associated with highest mortality rates even though incidence of infections by other related species is on the rise world over. Since *C. albicans* and other Candida species differ in their susceptibility to antifungal drug treatment, it is crucial to accurately identify the species for effective drug treatment. Most diagnostic tests that differentiate between *C. albicans* and other Candida species are time consuming, as they necessarily involve laboratory culturing. Others, which employ highly sensitive PCR based technologies often, yield false positives which is equally dangerous since that leads to unnecessary antifungal treatment. This is the first report of phage display technology based identification of short peptide sequences that can distinguish *C. albicans* from other closely related species. The peptides also show high degree of specificity towards its different morphological forms. Using fluorescence microscopy, we show that the peptides bind on the surface of these cells and obtained clones that could even specifically bind to only specific regions of cells indicating restricted distribution of the epitopes. What was peculiar and interesting was that the epitopes were carbohydrate in nature. This gives insight into the complexity of the carbohydrate composition of fungal cell walls. In an ELISA format these peptides allow specific detection of relatively small numbers of *C. albicans* cells. Hence, if used in combination, such a test could help accurate diagnosis and allow physicians to initiate appropriate drug therapy on time.

## Introduction

*Candida albicans* is an opportunistic human fungal pathogen of the gastrointestinal and genitourinary tracts [1]. Infections with *C. albicans*, a commensal in healthy individuals is associated with immunocompromised conditions in patients and delayed treatment of systemic infections can lead to increased mortality rates [2]. Early initiation of anti-fungal drug therapy depends on proper diagnosis of the pathogen. For routine identification of *Candida* isolates, conventional methods including germ tube formation, chlamydospore formation and sugar assimilation tests are universally accepted [3]. However, except for germ tube formation, other culturing techniques are time consuming, and can take up to 3 weeks. In immunocompromised patients, the clinical appearance of *C. albicans* infection is often very complex and identification of the species is difficult [4]. Lack of a reliable early diagnosis can lead to unnecessary arbitrary treatment of patients with antifungals that are toxic and expensive [5]. Many groups have developed PCR based assays for detection and identification of medically important yeast species [6], [7]. However, these methods which claim high sensitivity also require greater technical skills as well as expensive instrumentation and are likely to yield false positive results. In such a scenario, rapid identification methods using simple ELISA could be more useful compared to expensive PCR based assays or time consuming traditional culture based methods.

Stable short peptide sequences find their use as antimicrobial agents against many pathogens [8]. It is reported that histidine-rich peptides called Histatins are present in human parotid and sub-mandibular saliva. Histatins mediate killing of *C. albicans* by causing leakage of intracellular potassium ions and ATP [9], [10]. Similarly, other short peptides which specifically bind to the cell surface of *C. albicans*, may find important place in the area of diagnosis. Here, we present first report of identification of 12 mer peptides derived from a phage displayed peptide library selected against the filamentous form of *C. albicans*. We show that these peptides can successfully distinguish *C. albicans* from six other closely related *Candida* species and propose that they can be used as reagents in diagnostic tests.

## Results

### Phage display selection allowed identification of several *Candida albicans* specific clones

**Figure 1:**
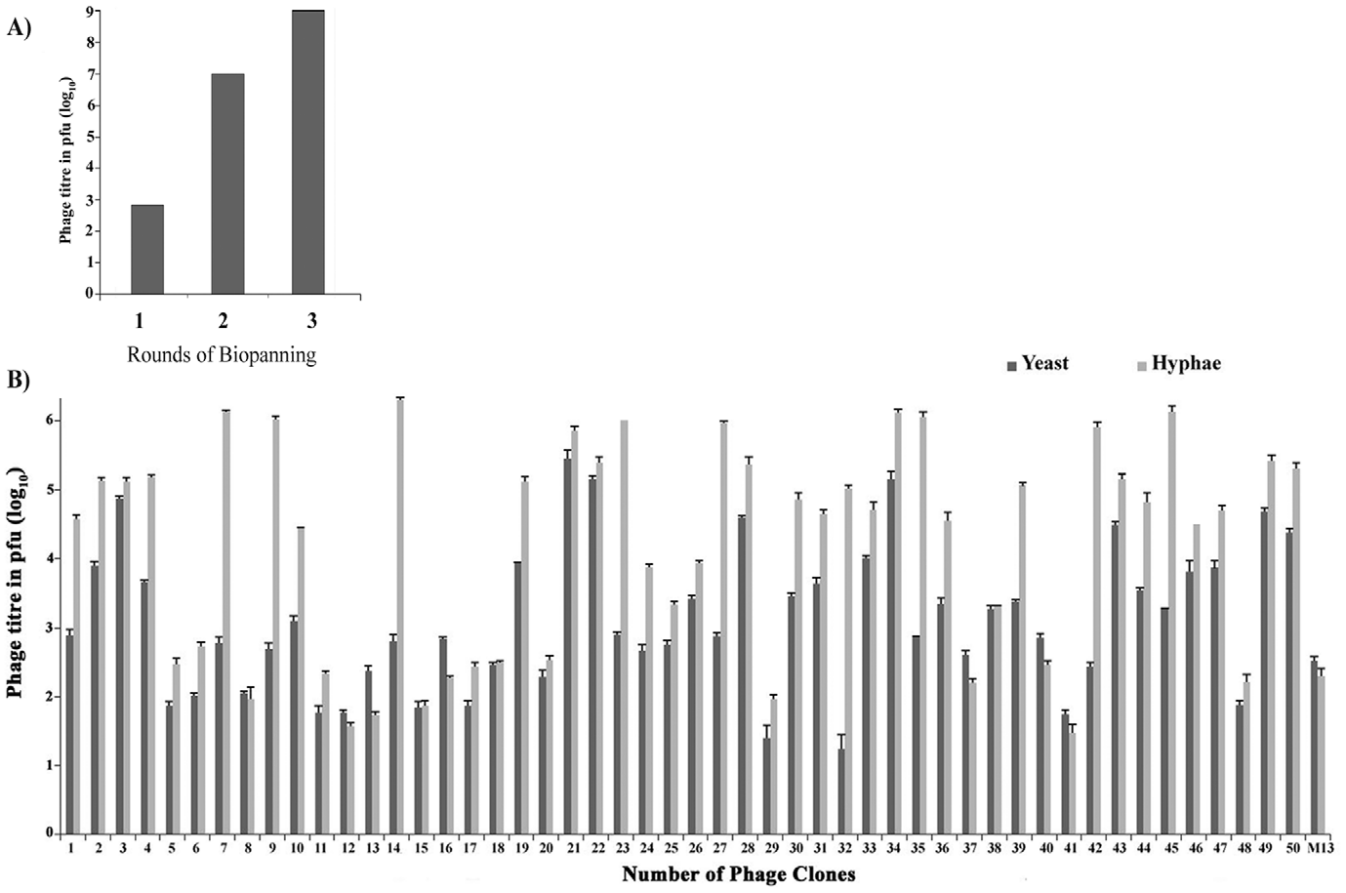
Selective enrichment of phages through biopanning. A) Phage peptide library against *C. albicans* hyphal cells after 3 rounds of bio panning. Gradual increase in phage titres was observed during first (6.8×10^2^ pfu), second (1×10^7^ pfu) and third round (1×10^9^ pfu) of bio panning. B) Plaque assay to determine binding specificity of clones to yeast (black bars) or hyphal (grey bars) form cells. Y axis represents the number of phages bound to 5×10^6^ cells. M13 phage without peptide served as a negative control.

As described above, three rounds of biopanning were performed and enrichment level was monitored after each round by determining the titre of eluted phages using plaque assay. As shown in Figure 1A, at the end of three rounds, a gradual increase in the phage titre led to effective enrichment of the order of 10^6^. Such high enrichment indicated that a small number of phages that specifically interact with *C. albicans* cells are highly represented in the bound phages. We isolated individual phage clones and initiated further characterization. A total of 50 well isolated plaques (labelled as H-1 to H-50) were picked and amplified to 2×10^11^ pfu/ml.

### Differential binding of phage clones to yeast versus hyphal cells of *C. albicans*

Although the phage clones were selected for binding against hyphal form of *C. albicans* cells, some of the clones might have ability to bind the yeast form as well, since cells induced to form hyphae are associated with their mother cells which are in the yeast form. There are also some small percent of yeast cells which have not undergone hyphal transition. Hence we decided to determine the binding affinity of selected phage clones towards the two morphological forms. The phage titre after interaction with equal number of cells either in yeast form or hyphal form reflected differences in affinity of binding between the different phage clones and the specific *C. albicans* morphological form. Out of 50 phage clones, H-34 showed high level of specific binding to both yeast and the hyphal form of cells compared to M13 phage displaying no peptide (Fig. 1B). We observed significant variation in the affinity of various phage isolates. Seven clones (H-7, 9, 14, 23, 27, 35 and 42) showed very high level of specificity towards hyphal cells as compared to yeast cells, while sixteen clones (H-5, 6, 8, 11, 12, 13, 15, 16, 17, 18, 20, 29, 37, 41, 45 and 48) showed low level of affinity with either yeast/hyphal cells with titre values not significantly different than M13 phage. Remaining 27 clones showed 10 to 100 fold higher binding with yeast or hyphal cells as compared to M13. These were considered as moderately specific clones and pursued further for detailed analysis along with the 7 hyphal specific clones.

### Sequencing of selected phage clones showed enrichment of a few peptide sequences

Out of these 50 clones, 34 positive phage clones (7 highly hyphal specific and 27 moderately specific to either yeast/hyphal form) and 2 clones (H-15 and H-41) showing no significant binding, were used to isolate single-stranded DNA [11]. The region of the phage genome (N-terminal of g-III region) that encodes the inserted 12-mer peptide was sequenced and the amino acid sequence of the peptide was deduced for each clone. The amino acid sequences revealed a total of 11 different peptide sequences, of which only 5 sequences were represented in multiple clones (Table 1). The consensus sequence “SE*****I*T**” predominated among the clones binding highly specifically to the hyphal cells, while “EL*A**I/V*****” was predominant in clones binding to hyphal cells only slightly better than the yeast cells. We selected 7 phage clones based on their differential binding abilities for further studies.

**Table 1:** Peptide sequences differ significantly with their ability to bind either yeast or hyphal cells.

No.	Sequences	Phage clones	Binding efficiency
1	**ELMAVPVPLPPA**	H01 ⊆ A	H≈Y
2	**SEYTSQLIFTAT**	H07 ⊆ B	H>>Y
3	**SEFSYIVIDTSL**	H09 ⊆ C	H>>Y
4	**ELTAILVSPAPL**	H22⊆ D	H≈Y
5	**ELNAQHIMEPKY**	H49 ⊆ E	H≈Y
6	**ELIPMLIMQSTS**	H46	H≈Y
7	**EDYSTIMKTLAH**	H25	H≈Y
8	**STPKSPHSVASH**	H38	H≈Y
9	**AVQHNPTHPFYP**	H40	H≈Y
10	**AHSTGLSPSTLR**	H15	Poor binders
11	**TMSSVAPRNLSS**	H41	Poor binders

>>, Highly specific; ≈, moderate binding. H, Hyphal cells; Y, yeast cells.

(⊆- subset;Group AH-01, 24, 32, 36, 44; Group B- H-07, 14, 23; Group C- H-09, 27, 35, 42; Group D- H-03, 19, 21,22, 28, 31, 34, 43; Group E- H-02, 04, 10, 26, 30, 33, 39, 47, 49, 50).

### The peptides confer high specificity towards *C. albicans* cells

**Figure 2:**
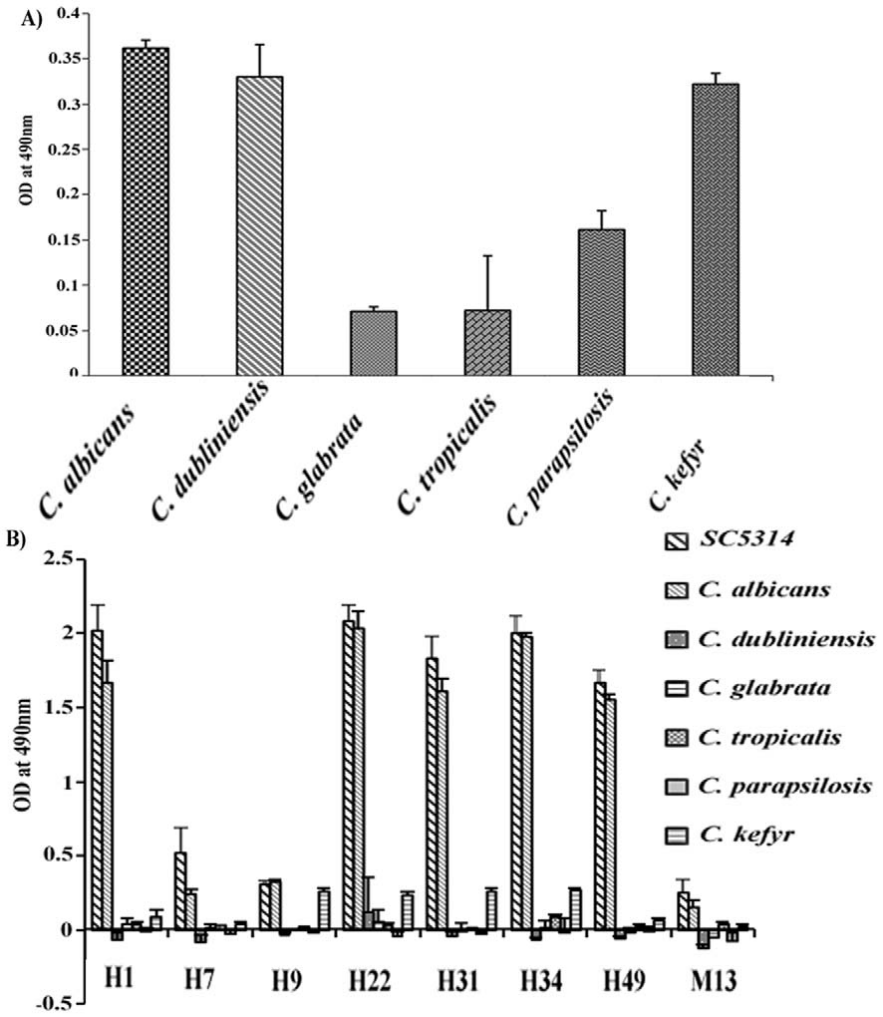
Interaction of different Candida species with A) commercially available polyclonal anti *C. albicans* antibody or B) phage clones. Only yeast form of cells of the indicated Candida species was used. A_490_ values signify the strength of interaction.

Since the present study was initiated with the objective of developing a simple assay for detection of *C. albicans*, we decided to use these phage clones for development of an ELISA and compare it with ELISA using a commercially available antibody raised against *C. albicans*. Since most other related species do not readily form hyphae under disease condition, it is challenging to distinguish *C. albicans* from other *Candida* spp., in its yeast form. Hence, we used yeast cells of *C. albicans*, *C. dubliniensis*, *C. glabrata*, *C. kefyr*, *C. parapsilosis* and *C. tropicalis* to test the specificity of the phage clones. The microtitre wells were coated with 5×10^6^ cells of the Candida species in yeast form, blocked with 2% BSA for 1 hour at 37°C and the ELISA was carried out using the commercially available polyclonal anti *C. albicans* antibody. The colour developed with OPD/H_2_O_2_ as substrate indicated the strength of interaction. Surprisingly, this antibody showed interaction with all the *Candida* species tested. *C. albicans*, *C. kefyr*, and *C. dubliniensis* cells showed greater reactivity to the antibody, than the *C. tropicalis*, *C. glabrata* and *C. parapsilosis* with as high as 5×10^6^ cells (Fig. 2A).

Subsequently, we performed ELISA using 7 phage clones described above, including hyphal form specific clones H7 and H9; as well as both yeast and hyphal form interacting clones H1, H22, H31, H34 and H49. Since the *Candida* cells coated were in the yeast form, except for the hyphal specific clones #7 and #9, all other clones showed several fold higher level of binding to *C. albicans* as compared to all other species (Fig. 2B). The M13 phage without any peptide insert showed minimal or no binding. Monitoring both the germ tube formation and growth on CHROMagar we confirmed that the species used showed expected phenotypes e.g. only *C. albicans* and *C. dubliniensis* formed germ tubes at 37°C in the presence of serum with significant quantitative difference in germ tube formation between *C. albicans* and *C. dubliniensis* (data not shown). All the *Candida* species after incubation at 37°C for 48 hours showed distinct colony colour on CHROMagar, specific to the respective species as reported earlier [12]. All these results confirmed that our phage clones can specifically identify *C. albicans* even in comparison to a closely related species like *C. dubliniensis.*

### The *C. albicans* specific clones show specific binding even in presence of several fold high level of competing *C. dubliniensis* cells

**Figure 3:**
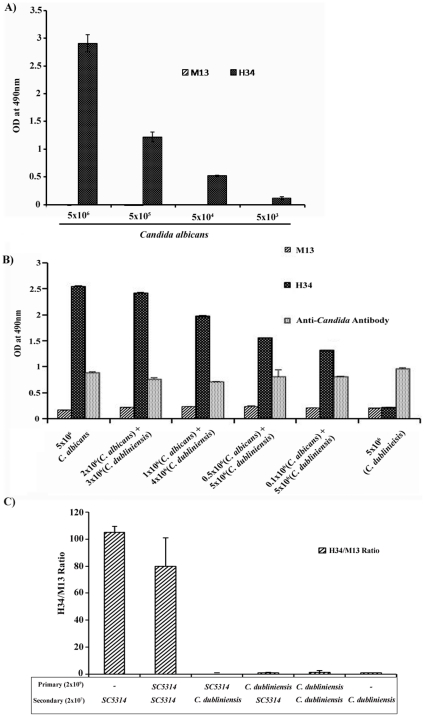
The phage clone can specifically detect low numbers of *C. albicans* cells. A) *C. albicans* cells were serially diluted from 10^6^ to 10^3^ and coated on the microtitre plate. M13 was used as a negative control. B) *C. albicans* cells were mixed with *C. dubliniensis* in different ratios keeping the total number of cells coated to be ∼5×10^6^ per well. The actual number of each type of cell is indicated below each bar. Anti *Candida* antibody which detects both species was used as a control to test for overall equal number of cells coated. C) Primary binding of 10^9^ phages with 10^6^ cells was estimated by plaque assay. The bound phages after elution were directly interacted with either *C. albicans* or *C. dubliniensis* during secondary interaction. Ratio of bound phages of H34 to M13 was determined during either primary or secondary interaction. Cells were visualized at 800X magnification.

The mortality rate in immunocompromised patients could be reduced by detection of lower number of *C. albicans* cells at an early stage of infection. The sensitivity of ELISA was tested with *C. albicans* cells ranging from 5×10^6^ to 5×10^3^ interacted with 2×10^7^ phages (H34). We observed more than 10 fold reduction in absorbance at 490 nm and there was no detectable interaction with less than 5×10^3^ cells (data not shown). This showed that in the current form, the ELISA could detect upto 5×10^3^ cells (Fig. 3A). M13 was used as a negative control which showed no detectable interaction with the *C. albicans* cells.

Since we showed that the clones selected above could bind specifically to *C. albicans* cells, we decided to test the specificity of interaction in more stringent conditions. We mixed the *C. albicans* cells with the closely related species, *C. dubliniensis* in several different ratios and then carried out the ELISA test as described above. Even at the ratio of 50∶1 *C. dubliniensis: C. albicans* cells, we observed highly specific binding of the H34 clone with the *C. albicans* cells in the yeast form. Thus, we could easily detect as low as 1×10^5^ cells (Fig. 3B). Further reduction of *C. albicans* cells showed overall reduced level of interaction (data not shown) and we suspect that the binding of cells was potentially due to inefficient binding of the *C. albicans* cells to the well surfaces used for ELISA.

Yet another way to confirm the specificity was to test if the bound phages continue to show species specificity after elution which should show enrichment of phages. We tested this by first interacting H34 phage clone with either of the species, eluted the bound phages and allowed them to interact with the cells of both the species again. As before, we saw that clone H34 binds specifically to *C. albicans* but do not bind to *C. dubliniensis* cells (Figure 3C). However, there was no significant interaction with *C. dubliniensis* either in the first or second round.

### Immunofluorescence assay confirms surface binding of the phage clones

**Figure 4:**
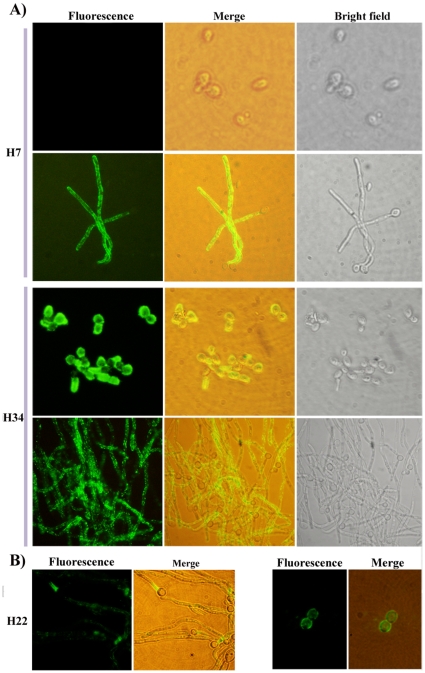
Immunofluorescene assay shows morphology specific binding of phage clones. A) The clone H7 can distinguish between the yeast mother cells and the hyphae emanating from them while clone 34 exhibits equal binding with both yeast and hyphal form of *C. albicans*. B) Clone 22 binds to specific regions on the surface of *C. albicans* cells. The fluorescent image and the merge of fluorescent and brightfield image are shown for cells induced to form hyphae (left panel) and yeast cells (right panel).

To support the observations reported above through ELISA, we tested the ability of some of these clones to bind to the yeast Vs hyphal cells using immunofluorescence assay. We incubated the cells in either yeast or hyphal form independently, with the phage clones and performed immunofluorescence assay as described in the materials and methods section. Figure 4A shows representative clones which showed specific interactions with yeast or hyphal cells. We saw that clone H7 showed only hyphal specific staining such that even the mother cells from which hyphae had emerged, showed no staining; on the other hand, H34 clone bound with both yeast and hyphal forms equally well (Fig. 4A) thus confirming the specificity of interaction with the morphological forms. Interestingly, after hyphal induction the H22 clone showed intense staining of a region of hyphae close to the mother cell and weaker staining over the rest of the hyphal surface while the mother cells showed almost no staining (Fig. 4B; left panel). The yeast form cells however showed a patchy staining (Fig. 4B; right panel). This indicated that during hyphal induction H22 binding epitopes may be getting redistributed over different regions of the cell surface.

Since it is likely that one could encounter *C. albicans* in blood samples, to test if we could detect the cells in a sample mimicking the samples in the field, we mixed the *C. albicans* cells in blood (see Materials and Methods) and then smeared the samples on to glass slides. After performing immunofluorescence study as described above, we could specifically detect the *C. albicans* cells without any other background (Figure S1). This suggested that our reagents will not react with other components of mammalian blood.

### The H34 clone binds a carbohydrate moiety on the cell surface on *C. albicans* cells

**Figure 5:**
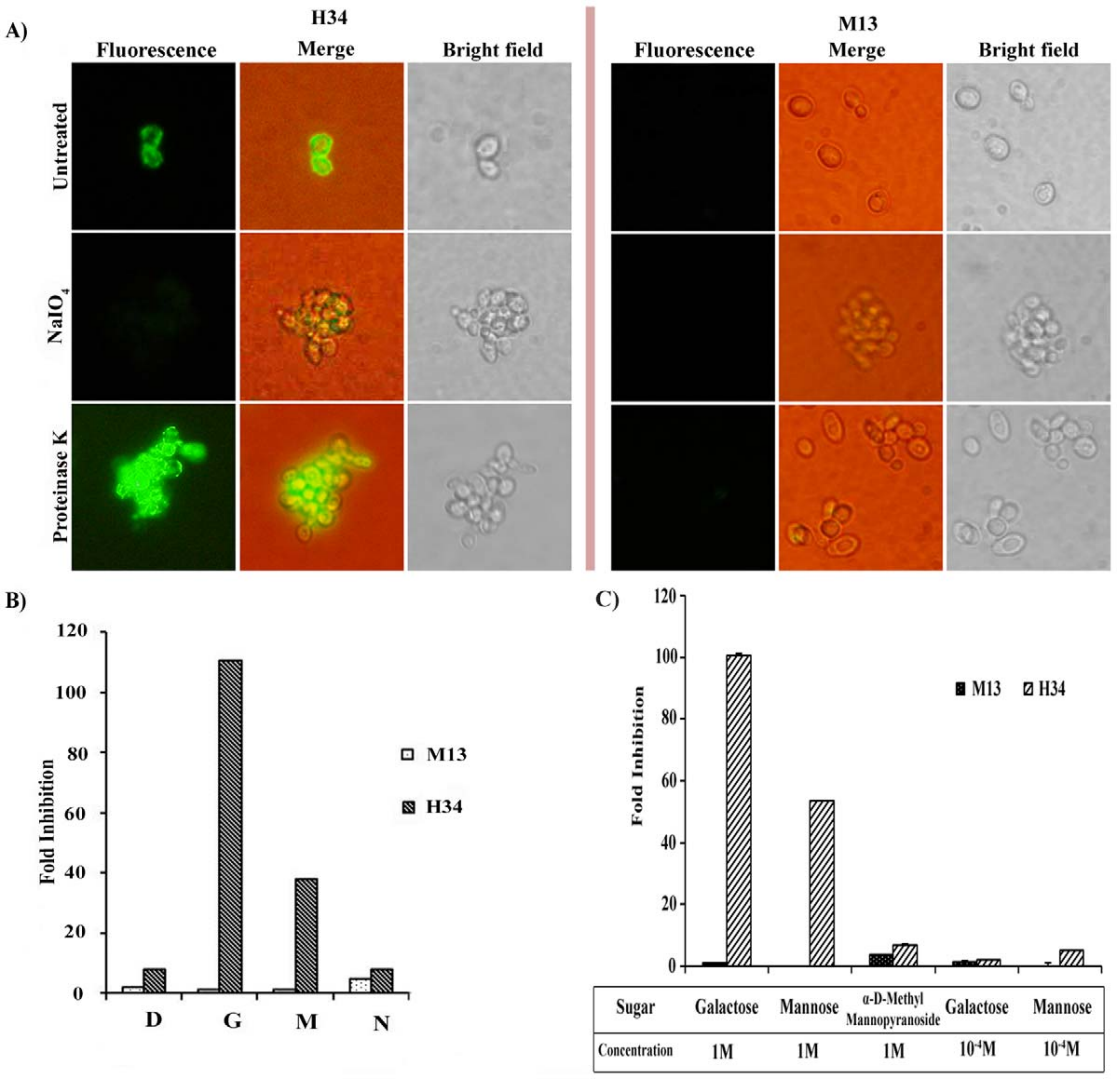
Biochemical characterization of cell surface ligands. A) *C. albicans* cells treated with proteinase K or NaIO_4_ followed by interaction with H34 clone showed binding insensitive to the proteinase treatment. M13 used as a negative control showed no interaction with untreated or treated cells. Cells were visualized at 800X magnification. B) Presence of excess of some monosaccharides inhibits the peptide- cell interactions. Phage clones incubated with excess (1 M) of Glucose (D), Galactose (G) Mannose (M), and N acetyl glucosamine (N) were allowed to interact with *C. albicans* yeast cells and the extent of interaction (or fold inhibition) was determined by plaque assay. C) Inhibition by excess monosaccharides is concentration and isomer dependent. In the same assay as in B, Mannose or α−D–methyl mannopyranoside used at 1 M and galactose or mannose at 10^−4^ M concentration and inhibition was determined as above. Values shown represent an average of three readings.

Since the phages showed specific binding on *C. albicans* cell surface we tested if the specificity of binding could be attributed to carbohydrate or proteinacious moieties on the cell surface. We treated the cells with either sodium metaperiodate or proteinase K, as described above and then performed immunofluorescence assay using clone H34. We observed that proteinase K treated cells continued to show fluorescence associated with the cell surface, while sodium metaperiodate treated cells showed no fluorescence at all, indicating complete loss of phage interaction (Fig. 5A). M13 phage without peptides showed no fluorescence associated with either untreated or treated cells.

To check further if excess specific monosaccharides will inhibit the specific binding of phage clones, we performed the sugar inhibition assay as described in the materials and methods section. We could see high level of inhibition (upto 100 fold) with 1 M of galactose and mannose but only a moderate level of inhibition with dextrose (Fig. 5B). We observed no inhibition with 1 M N acetyl glucosamine, a component of chitin, the innermost layer of the fungal cell wall. We also saw no inhibition with either α-D-methyl mannopyranoside, or lower concentration (10^−4^ M) of galactose or mannose (Fig. 5C). In all these experiments M13 binding was not affected by presence of excess of these carbohydrates.

Thus we conclude that the peptide in clone H34 specifically bound to some carbohydrate moiety which either has galactose or mannose as one of the components on the cell wall of *C. albicans* cells.

## Discussion

Phage display technique has proved to be a versatile and reliable tool for selecting short peptides against many biological or non biological targets [13]. With the rapid development of the technique over the past decade, phage display has been used successfully in numerous applications, including antibody engineering [14], peptide and protein drug discovery and manufacture [15], vaccine development [16] and identification of ligands [17]. Phage display screen has been reported against whole cells as well as against whole viruses [18],[19].

In the present study, we successfully used phage library displaying 12 mer random peptides to select *C. albicans* specific peptides. While two of these were highly specific to the hyphal form of cells, 5 peptide clones, showed comparable level of binding with both yeast and hyphal cells. Of course it is important to remember that hyphal cells provide much larger surface area for binding than the same number of yeast cells. Hence the level of hyphal binding as indicated by the ELISA may be an over estimate in favour of the hyphal cells. It is not inconceivable to imagine that there are identical epitopes present on both yeast and hyphal cells. It shall be noted that, these peptides do not bind to any other *Candida* species including *C. dubliniensis*, which closely resembles *C. albicans* with respect to genome composition and phenotypes. This indicates that the peptides bind to epitopes highly specific to *C. albicans*, thus making these peptides unique and versatile reagents for diagnosis of *C. albicans*. We have determined that at least some of these highly specific epitopes are carbohydrate in nature. It was interesting that the carbohydrate moiety detected by the phage clone H34 at least partially had galactose or mannose as one of its component. Glucose which is structurally closely related but distinct could inhibit only to moderate level. It is well documented that the yeast and hyphal cell walls differ in composition of mannan which form the outermost layer of the cell wall in *C. albicans.* It is not surprising that the epitopes differ among different species, which might reflect subtle differences in carbohydrate metabolism and cell wall biogenesis in these related Candida species.

For convenient identification of *Candida* spp. germ tube and chlamydospore formation as well as sugar assimilation tests, have been used for a long time [20]. In addition to these conventional methods, molecular methods including PCR based amplification of highly conserved sequences in the genome is also reported [6], [7]. Only germ tube formation test is widely employed in most of the clinical laboratories for rapid identification of *C. albicans*. However, it has been reported that *C. dubliniensis*, *C. parapsilosis*, *C. tropicalis* can also form germ tubes/pseudohyphae in serum and their sugar assimilation patterns are similar to that of *C. albicans*, hence chances of incorrect diagnosis are high without a test that can unequivocally differentiate between these related species in a short time [4].

The two species *C. albicans* and *C. dubliniensis* are highly similar with respect to their genome sequences and exhibit high similarity in many phenotypic characters including resistance to cycloheximide, ability to produce germ tubes, chlamydospores and true hyphae [21]. Although sugar assimilation, growth at elevated temperature and intracellular β-glucosidase activities differ between these two species [22], [23], none of these methods have proved to be effective in differentiating the two species in clinical settings [24]. On the other hand, no single molecular fingerprinting technique has evolved as a dominant method, as each method has its own set of limitations [25], [26]. Most PCR based methods often turn out to be too sensitive and while they claim to be able to detect as low as single cell per ml, even degradation products from dead cells can lead to false positives in these tests. Bliss et al. (2003) reported identification of ScFv expressing phage clones that could differentiate between filaments formed by these two species [27]. These reagents were specific to proteins expressed on the filaments by either of the species. In contrast, some of the peptides reported in the present study could detect specific carbohydrate moieties on the surface of *C. albicans* irrespective of the morphological form while others could specifically detect ligands on the hyphal form of *C. albicans*. Some of the peptides curiously could even decorate specific domains on hyphae e.g. clone H22 which stains heavily on surface of hyphae close to the mother cell. While the clones thus can be used in a diagnostic test, studying specificity of interaction of H22 will serve an academic interest, since it can differentiate different regions of surface of *Candida* hyphae. Hence, not only can these peptides be used in combination for developing highly sensitive diagnostic tests, they can be used in studying finer qualitative differences in surface carbohydrates of these fungal pathogens which could contribute differently to the host pathogen interactions. These peptides can be employed for rapid detection of *C. albicans* by a simple ELISA based assay which allows testing multiple samples at the same time and thus making the assay high-throughput.

Among systemic fungal infections, 60% are caused by *C. albicans* and there is an increase in the number of cases of infection due to non-albicans species like *C. tropicalis*, *C. parapsilosis*, *C. glabrata* and *C. krusei* [5]. Since these species differ in their antifungal susceptibilities and the cost of antifungal treatment is high, it is imperative to make accurate and timely diagnosis of the causative agent. Therefore, it is crucial to develop species specific diagnostic test for all of these species and we believe that, this can be possible with short peptides identified by phage display technique as reported here.

## Materials and Methods

### Candida strains, culture and experimental condition

Clinical isolate of *C. albicans*, SC5314 [28] was used for phage peptide library screening. Other *Candida* species used for comparative binding studies were *C. glabrata* (Dr Rupinder Kaur, CDFD, Hyderabad), *C. dubliniensis*, *and C. tropicalis* (Dr Kaustuv Sanyal, JNC, Bangalore), *C. albicans*, *C. parapsilosis* and *C. kefyr* (Dr. Y Prakash, KMC, Manipal). Candida strains were routinely grown in YEPD broth (1% Yeast extract, 2% Peptone, 2% Dextrose) overnight at 30°C. Under these conditions all Candida strains grow extensively as yeast cells. CHROMagar (Difco) was used for identification of Candida species.

### *C. albicans* hyphal induction

For hyphal induction, 5×10^6^ yeast cells of *C. albicans* were incubated in synthetic complete drop-out medium [29] supplemented with 4% glucose and 10% fetal bovine serum (FBS; Gibco-BRL) at 37°C for 4 hours on a rotary shaker.

### Phage libraryand biopanning of phages binding specifically to *C. albicans*

Phage library used in this study, contained M13 phage clones displaying 12 mer random peptides fused to pIII coat protein (New England Biolabs). Biopanning and amplification of the phages was performed according to the procedure recommended by the manufacturer. In brief, 5×10^6^ *C. albicans* cells induced to form hyphae, were blocked with 2% BSA for 2 hours at room temperature (RT) and later washed with Tris buffer (TBS pH 7.3) in 1.5 ml eppendorf tubes. Phage library was diluted in blocking buffer (TBS pH7.3, 2% BSA) and 10^11^ pfus of the phage library were co-incubated with ∼5×10^6^ hyphal cells at RT for 2 hours on a rotator. Unbound phages were washed with TBST buffer (TBS pH 7.3, 0.1% Tween 20). The bound phages were eluted with 80 µl of 0.1 M glycine (pH 2) for 10 min and neutralized with 20 µl of 1 M Tris buffer pH 8.0. The eluted phages were serially diluted and used to infect *E. coli* (ER-2738) and plated on LB^Tet^ plates to estimate the titre values. After first round of panning, eluted phages were amplified to a concentration of 2×10^11^ pfu/ml and again used as input for second round of biopanning. Second and third round was each performed in the same way as the first round, except that 0.3% and 0.5% Tween-20 was used respectively during washing step to increase the stringency of binding of peptides to the cells. After the third round of selection, individual plaques on LB plates were picked with sterile tooth pick and maintained as individual phage clones for further studies.

### Assays for detection of peptide dependent phage binding to *C. albicans* cells

#### Plaque assay

Binding studies of individual phage clones with *C. albicans* cells (both yeast and hyphal form) were performed to select phage clones which show specific binding. Binding of individual phage clones was confirmed by plaque assay performed in triplicate. In brief, 5×10^6^ cells of *C. albicans* in either yeast or hyphal form, were mixed with a single phage clone having 2×10^9^ pfu/ml phages in an eppendorf tube and incubated at RT for 1 hour. Unbound phages were removed by three washes with TBST buffer (0.5% Tween 20 in TBS) followed by two washes with TBS. Bound phages were eluted with 80 µl of 0.1 M glycine (pH 2.0) for 10 min and immediately neutralized with 20 µl of 1 M Tris buffer pH 8.0. Phage titre was estimated as above. In all experiments M13 phage without displaying any peptide was used as negative control.

Specificity of H34 phage clone was further conformed by two rounds of plaque assay against 5×10^6^ cells of *C. albicans* or *C. dubliniensis* as mentioned above. In the first round, 2×10^9^ pfu/ml phages were used for the interaction, whereas in the second round, the eluted phages obtained from the first round estimated as 2×10^7^ pfu were used. The plaque assay in which 5×10^6^ cells of *C. albicans* or *C. dubliniensis* were interacted with 2×10^7^ pfu/ml phages used as a control and M13 phage was used as a negative control.

#### ELISA

Phage clones were further tested by ELISA for their ability to bind specifically to C. albicans. All ELISA tests were performed in triplicate in 96 well poly-lysine coated microtitre plates. All wells were incubated with 100 µl of 5×107/ml yeast cells of C. albicans at 37°C for 1 hour. After decanting, wells were blocked with 2% BSA for 1 hour at 37°C and then 2×107 pfus of individual phage clone in 100 µl of TBS were added to each well. After 1 hour incubation at RT on orbital shaker, wells were washed once with 0.5% TBST and incubated for 1 hour with 100 µl of mouse antiM13 horseradish peroxidase (HRP) conjugate (GE Healthcare, Munchen, Germany: 1∶15000) at RT on orbital shaker. The wells were washed once with 0.1% TBST and presence of HRP was detected with o- phenylene diamine (OPD)/H2O2 as substrate. The reaction was stopped by addition of 50 µl of 2.5 M sulphuric acid. Absorbance at 490 nm was measured by using ELISA plate reader (SPECTRAmax 340).

As control ELISA was performed using rabbit polyclonal anti *C. albicans* antibody (Takara Bio Inc), as primary antibody (1∶2,000), instead of phage clones and anti rabbit HRP conjugate (1∶15,000) (Sigma) was used as the secondary antibody to detect the primary antibody bound to Candida species cells. Peroxidase level was determined as described above.

#### Competitive ELISA

To check the sensitivity and specificity of the assay, we mixed the C. albicans and C. dubliniensis in different ratios (numbers) as well as C. albicans alone coated on the well and performed the ELISA experiment as described above.

#### Immunofluorescence assay

Yeast form of C. albicans cells were spotted and incubated at 37°C for 1 hour on poly lysine coated glass slides. Non specific binding sites were blocked with 2% BSA for 1–2 hours at 37°C. Cells were spotted and incubated with 2×109 pfu of specific phage clone in 100 µl of TBS for 1 hour at RT on rocker. Phages were washed off once with 0.1% TBST and cells were incubated with HRP conjugated mouse anti M13 antibody (1∶100 dilution) at RT. After 1 hour incubation, cells were washed once with TBS and incubated with 1∶1000 dilution of anti-mouse antibody-FITC conjugate for 1 hour on rocker at RT. Slides were rinsed with TBS and observed using Olympus B51 fluorescence microscope.

Yeast form of *C. albicans* cells in the presence of blood were spotted on poly lysine coated glass slide and incubated at 37°C for 1 hour. The debris was removed by washing with 1X TBS. Cells were interacted with 2×10^9^ pfu of specific phage clone in 100 µl of TBS for 1 hour at RT on rocker and immunofluorescence was performed as described above.

#### Sugar inhibition assay

To test whether the binding of phage clones on involves interaction with sugar residues the cell wall of C. albicans, we performed the interactions in presence of high concentration of various monosaccharides. The phage clones (2×107 pfu) were mixed with 1 M of dextrose, galactose, mannose or N-Acetyl glucosamine in an eppendorf tube for 1 hr at RT. Then 5×106 C. albicans cells were added to the tube for interaction and the plaque assay was performed as described above. M13 phage was used as a negative control. The above experiment was performed with 1 M of α-D-methyl mannopyranoside, 10-4 M of galactose or mannose.

#### Biochemical analysis of peptide binding specificity

Overnight saturated C. albicans cells were washed with TBS and treated with either proteinase K to degrade proteinaceous antigens or with sodium metaperiodate to degrade carbohydrate antigens on the cell surface as described previously [27]. Briefly, for proteinase K treatment cells were incubated in 50 mM Tris (pH 7.5) containing 100 µg/ml of proteinase K for 2 h at 37°C. For sodium metaperiodate treatment, cells were incubated in 50 mM sodium acetate (pH 4.5) buffer containing 50 mM sodium metaperiodate for 30 min at 4°C in the dark. After washing with water, treated cells were analyzed by immunofluorescence as described above.

## Supporting Information

Figure S1 **Immunofluorescence assay shows that Candida specific phages do not react with components of mammalian blood.** As described in the methods section the cells mixed with rabbit blood were smeared and immunofluorescence assay was performed with H34 phage clone. Cells were visualized at 800X magnification. The brightfield image, the fluorescent image and the merge of the two are shown.
(TIF)
