# Supplementary figures and images for: Phage Displayed Short Peptides against Cells of Candida albicans Demonstrate Presence of Species, Morphology and Region Specific Carbohydrate Epitopes
Source: PLoS One. 2011 Feb 22;6(2):e16868. doi: 10.1371/journal.pone.0016868 (PMC3043061; doi:10.1371/journal.pone.0016868)

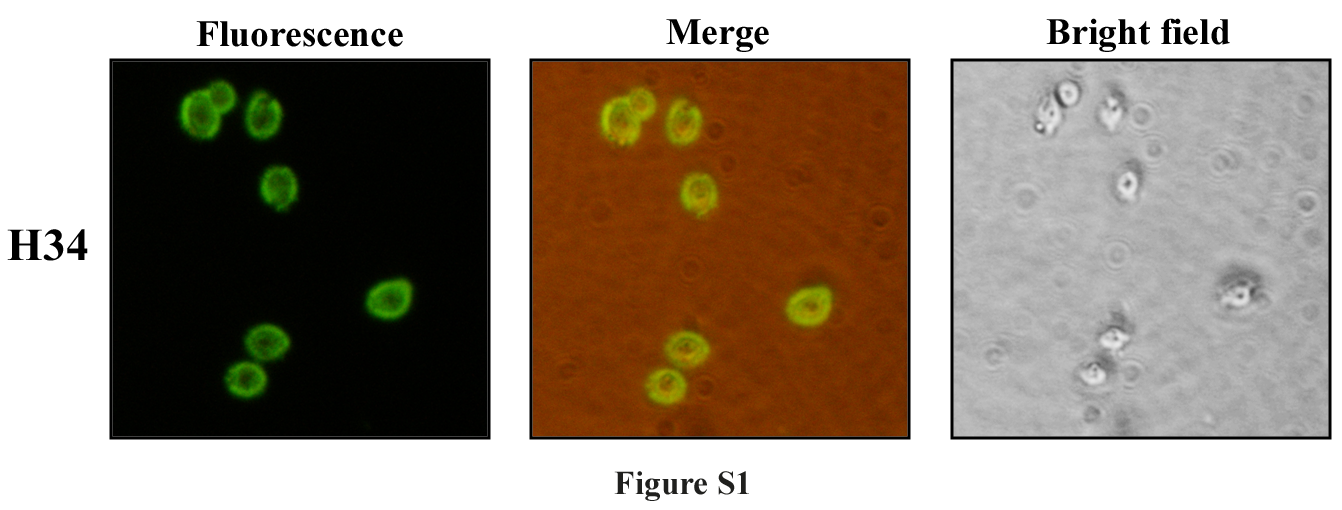

Supplement: Figure S1 — Immunofluorescence assay shows that Candida specific phages do not react with components of mammalian blood. As described in the methods section the cells mixed with rabbit blood were smeared and immunofluorescence assay was performed with H34 phage clone. Cells were visualized at 800X magnification. The brightfield image, the fluorescent image and the merge of the two are shown. (TIF) [file pone.0016868.s001.tif]
